# Dysrhythmogenic potential in acute admissions to psychiatric hospitals and clinics

**Published:** 2007-07

**Authors:** CC Grant, J Ker, M Viljoen, B Steenkamp, L Gauche, JL Roos, PJ Becker

**Affiliations:** Department of Physiology, University of Pretoria, Pretoria; Department of Physiology, University of Pretoria, Pretoria; Department of Physiology, University of Pretoria, Pretoria; Department of Psychiatry, University of Pretoria, Pretoria; Department of Psychiatry, University of Pretoria, Pretoria; Department of Psychiatry, University of Pretoria, Pretoria; Biostatistics Unit, Medical Research Council, Pretoria; Department of Clinical Epidemiology, University of Pretoria, Pretoria

## Abstract

**Summary:**

Co-morbidity between physical disease, especially cardiovascular, and psychological disturbances is well documented. In psychiatric patients, the potential for dysrhythmogenic incidences is increased by the fact that many psychiatric medications influence cardiovascular function.

**Aim:**

The aim of the study was to examine the dysrhythmogenic potential of 30 psychiatric patients (group A), irrespective of diagnoses or medication, at admission to psychiatric institutions.

**Methods:**

The dysrhythmogenic potential was determined in terms of heart rate-corrected QT intervals (QTc), heart rate-corrected JT intervals (JTc), QT and JT dispersion (QTcd and JTcd) between leads V1 and V6, and heart rate variability (HRV) as determined from lead V6 of the ECG. Values were compared with 30 age- and gender-matched controls (group B). In the second part of the study the dysrhythmogenic indicators were assessed in a patient group (group C; *n* = 43) with only psychiatric disorders and compared to a group with psychiatric as well as medical disorders (group D; *n* = 27).

**Results:**

The patient group A had significantly higher values than the control group for mean QTc (V6) (0.4579 ± 0.0328 vs 0.4042 ± 0.0326; *p* = 0.0470), mean JTc (V6) (0.3883 ± 0.0348 vs 0.3064 ± 0.0271; *p* = 0.0287) and mean QT and JT dispersion values (QTcd = 0.0443 ± 0.0203 vs 0.0039 ± 0.0053 and JTcd = 0.0546 ± 0.1075 vs 0.0143 ± 0.1450, *p* < 0.05). A statistically significant difference (*p* < 0.0001) was found between the patients’ (group A) HRV and that of the controls (group B). No statistically significant differences were found between the values of the dysrhythmogenic indicators for patients with only psychiatric illness (group C) and those with psychiatric as well as medical disorders (group D).

**Conclusions:**

Psychiatric patients at the point of admission to psychiatric institutions may have an increased dysrhythmogenic potential, not necessarily caused by physical disease. The potential of an augmented risk for cardiovascular incidents in psychiatric patients should be considered when treating such patients.

## Summary

Co-morbidity between physical disease and psychological stress or psychiatric disturbances is well documented. In general it would appear that negative emotions, be it through chronic activation of the hypothalamic−pituitary−adrenocortical axis, disturbances in the regulation of the autonomic nervous system, or upregulation of pro-inflammatory cytokine production, may have diverse adverse effects on health.^1^-^3^ One of the best-known associations is that between cardiovascular events and psychological stress where stress is implicated in the pathogenesis of cardiovascular disorders such as atherosclerosis, hypertension, myocardial infarction and sudden death.^4^ Whatever the causal link between cardiovascular risk and negative psychological phenomena, there is a growing awareness of this association, as evidenced by the INTERHEART study.^5^,^6^

In psychiatry, the existence of co-morbidity between depression and cardiovascular disorders is perhaps among the best known of these physical−psychological associations, and a host of articles has been published on this topic. Large meta-analytical studies are available in which publications on depression, either as risk factor for the development of myocardial infarction or cardiac death,^7^,^8^ or as a predictor of mortality in patients already diagnosed with coronary heart disease, were analysed.^9^,^10^

Although many questions, as well as some inconsistencies in the findings^11^ remain, there are firm grounds to assume that depression, or perhaps even depressed moods of sufficient duration, pose a risk to healthy subjects. Furthermore, depression in patients with established coronary heart disease is a risk factor for cardiac and all-cause mortality – a fact that cannot be ignored in patient care.^12^ Associations between the risk for cardiovascular incidents and other psychiatric disorders such as anxiety disorders have also been reported but the picture is less clear.^13^,^14^

If psychiatric disorders pose a threat to cardiovascular health, it is important that the fact should be recognised by clinicians working at psychiatric hospitals and clinics. It would be equally important that clinicians in general should be aware of the possibility of a higher risk when working with psychiatric patients. This is all the more important in view of the fact that many of the medications prescribed to psychiatric patients are known to influence cardiovascular function.^15^-^17^

This study examined the dysrhythmogenic potential of acute admissions to psychiatric hospitals by looking at QTc and JTc intervals, and QT and JT dispersion (between lead VI and V6) and heart rate variability (HRV) measured on lead V6 of the ECG. The aim was not to study possible links between specific psychiatric disturbances and cardiovascular health, but rather to assess the dysrhythmogenic potential of patients when they are admitted to psychiatric institutions, irrespective of psychiatric diagnoses or medication.

## Selection and description of participants

A major selection criterium was that patients who took part in the study conformed to the guidelines suggested by van Staden and Kruger for acquiring informed consent from psychiatric patients.^18^ Only patients who could understand and complete the informed consent form and from whom ECGs could be obtained, participated.

The total study population consisted of 70 consecutive, acute admissions to three psychiatric facilities. Diagnoses, medication, age, gender and weights were noted, but not used as inclusion or exclusion factors. Adult patients up to 30 years of age were included in the first part of the study. The control group comprised 30 healthy, active volunteers, who were age and gender matched to the patient group. Only people without psychiatric and cardiovascular disease, who were not using medication, and had normal arterial blood pressure were included in the control group.

In the first instance, the dysrhythmogenic potential of 30 patients (group A) was compared to 30 age- and gender-matched participants from the control group (group B). The total patient group (70) was then divided into psychiatric patients with and without physical disorders. The dysrhythmogenic potential of the 43 patients with psychiatric illness but no physical diseases (group C) was then compared to the dysrhythmogenic potential of the remaining 27 psychiatric patients (group D) who were presenting with psychiatric as well as physical diseases.

## Methods

This was a prospective, non-interventional, quantitative study where the individuals who analysed the recordings were blind to both the diagnoses and medications of the patients. The protocol was submitted to, and approved by, the Ethics Committee of the University of Pretoria, clearance no 31/2004. Standard 12-lead ECG recordings were obtained from all patients within 24 hours of admission to psychiatric institutions. ECGs were done with participants in the supine position, in a low-noise area, at a comfortable temperature (± 22°C).

The dysrhythmogenic potential was determined by assessing the following indicators obtained from 12-lead baseline ECG recordings:^20^,^21^

● heart rate-corrected QT intervals (QTc)
● QTc dispersion, ie, the difference between corrected QT times of lead V1 and V6
● heart rate-corrected JT intervals (JTc)
● JTc dispersion, ie, the difference between corrected JT times of lead V1 and V6
● heart rate variability (lead V6) by development of a discrete distribution of heart rate variability classes ranging from ‘no’ variability to ‘high’ variability.

The QT interval was determined as the time measured from the beginning of ventricular depolarisation to completion of repolarisation (from Q to T).^19^-^23^ The QT interval is commonly used as a measure of ventricular repolarisation and represents the time from the beginning of ventricular depolarisation to completion of repolarisation.^19^ During normal conduction, the QT interval is mainly determined by the repolarisation duration. However, in the case of increased QRS duration, the QT interval is said to have limited value because it includes ventricular depolarisation. It is therefore fairly generally accepted that measurement of the JT interval is a more appropriate measure of ventricular repolarisation than the QT interval.^20^,^21^

The JT interval was measured from the J-point to the end of the T-wave. To allow for variation in heart rate, heart rate-corrected QT and JT intervals (QTc and JTc) were calculated. The heart rate-corrected JT time was first calculated by dividing the JT time by the square root of the RR interval. These values (JTc for leads V1 and V6) were then used to determine the second indicator, ie, JT dispersion, an index of inhomogeniety of repolarisation.^20^,^21^ Similar calculations were done for the QT values.

## Measurements

● QTc, in seconds, was calculated as follows:^19^-^23^

$QTc\;V1:\;\;QT_{1}=\;\frac{QT}{\sqrt{RR}}$ measured on lead 1

$QTc\;V6:\;\;QT_{6}=\;\frac{QT}{\sqrt{RR}}$ measured on lead 6

QT dispersion was calculated from the measured QT times as follows:^24^,^25^

QT dispersion: ▵QT = QT_1_ - QT_6_

● JTc, in seconds, was calculated as follows:^19^-^23^

$JTc\;V1:\;\;JT_{1}=\;\frac{JT}{\sqrt{RR}}$ measured on lead 1

$JTc\;V6:\;\;JT_{6}=\;\frac{JT}{\sqrt{RR}}$ measured on lead 6

● JT dispersion was calculated from the measured JT times as follows:^24^,^25^

JT dispersion: ▵JT = JT_1_ - JT_6_

● The heart rate variability (HRV) was calculated by comparing the lengths of the intervals between four consecutive QRS complexes measured on lead V6 ECG recordings.^26^,^27^ The RR distances were measured and compared to one another. There were four different possible outcomes:

– the four RR times are the same: V6 variability = 0

– three RR times are the same: V6 variability = 1

– two RR times are the same: V6 variability = 2

– none of the RR times is the same: V6 variability = 3.

## Statistics

Thirty patients (group A) were compared to age- and gender-matched controls (group B) with respect to QTc, QT dispersion, JTc, and JT dispersion using the Mann-Whitney test, while the Fischer’s exact statistical test was employed to compare the groups with respect to HRV. The influence of the patients’ underlying medical conditions was determined by comparing QTc, QT dispersion, JTc, and JT dispersion from group C with those from group D by means of the Mann-Whitney test. The Fischer’s exact test was again employed to compare groups C and D with respect to HRV.

## Results

**Fig. 1.**
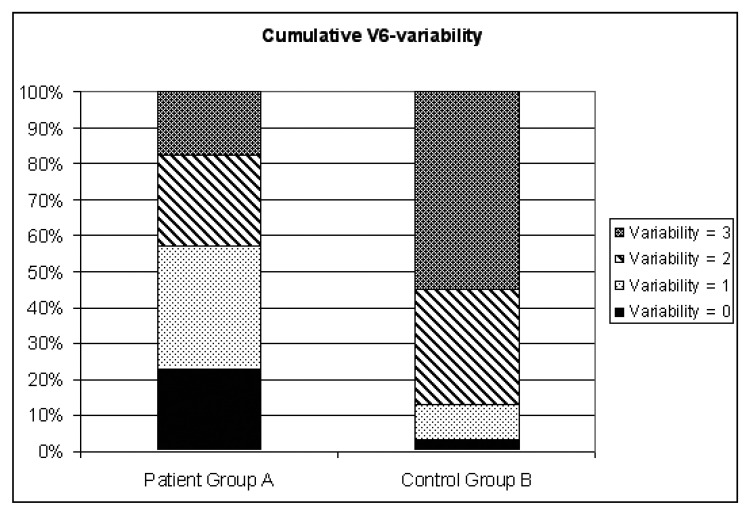
Distribution of RR variability for patient group A and control group B.

The demographic characteristics of the patient group relative to the control group are shown in Table 1. The mean values and standard deviations determined for groups A and B are shown in Table 2. A comparison of the HRV in groups A and B is shown in Fig. 1. A statistically significant difference (*p* < 0.0001) was found between the two groups.

**Table 1:** Demographic Data Of Patient Group A And Control Group B

	*Patient group A*						*Control group B*				
	*Age (years)*	*Height (cm)*	*Weight (kg)*	*Men*	*Women*	*Age (years)*	*Height (cm)*	*Weight (kg)*	*Men*	*Women*
Mean	27.1	1.72	71.2	16	14	26.6	1.74	72.1	16	14
SD	4.6	0.09	8.7			4.3	0.08	9.5		

**Table 2:** Comparison Of The QTc, QTcd, JTc And JTcd Between Group A (*n* = 30) And Group B (*n* = 30)

	*Group A*		*Group B*		
	*Mean*	*SD*	*Mean*	*SD*	p*-value*
QTc	0.4579	0.0328	0.4042	0.0326	0.0470*
QTcd	0.0443	0.0203	0.0039	0.0053	0.0000*
JTc	0.3883	0.0348	0.3064	0.0271	0.0287*
JTcd	0.0546	0.1075	0.0143	0.145	0.0460*
RR variability	NA				< 0.0001*

In the patient group, 23% indicated no heart rate variability; 34% showed low HRV; 25% showed medium variability and only 18% indicated high (normal) variability. In the control group, only 3.2% showed no HRV; 9.7% showed low variability; 32.3% was in the medium-variability category, while 54.8% had high variability.

Table 3 compares the patients with only psychiatric illness (Group C) to those with psychiatric as well as other medical illnesses (Group D). No differences were found between groups C and D for any of the indicators.

**Table 3:** Comparison Of The QTc, QTcd, JTc And JTcd Between Group C (*n* = 43) And Group D (*n* = 27)

	*Group C*		*Group D*		
	*Mean*	*SD*	*Mean*	*SD*	p*-value*
QTc	0.4041	0.0732	0.4371	0.0966	0.1092
QTcd	0.0440	0.0489	0.0592	0.0688	0.2842
JTc	0.3041	0.0733	0.3593	0.2046	0.1136
JTcd	0.0950	0.2070	0.1045	0.2273	0.8593
RR Variability	NA				0.1063

## Discussion

The study investigated the dysrhythmogenic potential in patients at admission to psychiatric hospitals. The aim of the study was neither to look at the co-morbidity between specific psychiatric disturbances and cardiovascular phenomena, nor to look at the influence of drugs, but rather to see if acute psychiatric admissions were more vulnerable to dysrhythmogenic incidents than individuals without psychiatric conditions. Dysrhythmogenic indicators determined from the ECGs included heart rate-corrected QT and JT intervals and QTc and JTc dispersion between lead V1 and V6, as well as heart rate variability determined on lead V6 recordings.

QTc intervals and dispersion as well as JTc intervals and dispersion are used as indicators in studies on repolarisation abnormalities where they appear to be effective in the identification of myocardial instability and therefore the risk for ventricular arrhythmias.^22^,^23^ Prolonged QT and JT intervals and an increase in dispersion are associated with arrhythmias and can provide prognostic information about future cardiac incidents. 20,22,23 An increase in inter-lead dispersion is known to be an indication of the inhomogeneity of ventricular repolarisation and is also linked to the onset of ventricular arrhythmias and sudden cardiac death.^24^,^25^ It is assumed that autonomic imbalance can change the repolarisation gradient from one beat to the next. This may account for the initiation of re-entrant ventricular arrhythmias under certain circumstances.^28^

In this study, the mean QTc of patient group A was significantly higher (0.4579 ± 0.0328) than that of the control group (0.4042 ± 0.0326) (*p* = 0.0470). Prolonged heart rate-corrected QT intervals are associated with increased mortality risk in patients with coronary heart disease and in the general population.^20^,^30^

In group A, the mean JTc value (0.3883 ± 0.0348) was also significantly higher than that of the control group B (0.3064 ± 0.0271), therefore indicating that the time-corrected repolarisation period of the patient group A was significantly longer than that of the control group B. This, by implication, indicated an increase in the absolute index of ventricular repolarisation − an electrographic parameter shown to be effective in the identification of electrical myocardial instability, and therefore risk for ventricular arrhythmias.^22^,^23^

QT and JT dispersion was calculated from the difference between the ECG lead V1 and V6. Results from the comparison of the patient group A and the age- and gender-matched control group B dispersion values are summarised in Table 2. The mean QTc and JTc dispersion values of the patient group A (QTcd = 0.0443 ± 0.0203; JTcd = 0.0546 ± 0.1075) were significantly higher (*p* < 0.05) than the mean values of the control group B (QTcd = 0.0039 ± 0.0053; JTcd = 0.0143 ± 0.1450).

The dispersion values of the psychiatric patient group therefore differed significantly from those of the healthy control group. No previously published articles were found on the dispersion values specific to psychiatric patients. However, several studies pointed out that there is an association between increased inhomogeneity of ventricular repolarisation and an increased risk of arrhythmias and sudden cardiac death.^24^,^25^,^28^,^30^ These results therefore support those of the other indicators in this study of an increased risk for ventricular arrhythmias.

The final dysrhythmogenic indicator investigated in the two groups was heart rate variability (HRV), ie, the beat-to-beat variation in heart rate around a mean value − which is modulated by the autonomic nervous system. A marked reduction in HRV is a strong predictor of sudden death following MI and arrhythmic complications.^29^ In this study, the RR intervals were measured on the ECG lead V6, and the participants were grouped together in classes of differing variability. The distribution of RR variability (lead V6) in the patient and control groups are summarised in Fig. 1.

A statistically significant difference (*p* < 0.0001) was found between the patient and control groups. In the patient group, 23% indicated no heart rate variability; 34% showed low variability; 25% showed medium variability and only 18% indicated high (normal) variability. In the control group, only 3.2% showed no variability; 9.7% showed low variability; 32.3% fell in the medium-variability category, while 54.8% had high variability.

Variability in the patient group centred around the medium-, low- and no-variability categories, while variability in the control group centred around the medium- and high-variability categories. Therefore, according to the RR variability (lead V6), the psychiatric patient group represented a significantly lower HRV category than the control group, thus indicating an increased risk of coronary heart disease and arrhythmias. This is in correspondence with results by Gormann *et al.* and Hughes *et al.*^14^,^26^

The comparison of dysrhythmogenic indicators between the patients with only psychiatric illness (group C) and those with psychiatric as well as physical illness (group D) indicated no statistically significant differences (*p* > 0.05). The values obtained are shown in Table 3. The implications are therefore that the difference in dysrhythmogenic potential cannot summarily be ascribed to underlying physical disorders.

## Conclusions

In this study, the potential dysrhythmogenic potential was assessed by means of heart rate variability, JTc times and JTc dispersion. The results support the indications of previous research, among others the INTERHEART study,^13^-^17^,^26^,^27^,^31^ that psychiatric patients may have an increased dysrhythmogenic potential. This is in line with the assumption that an increased dysrhythmogenic potential in psychiatric patients can, theoretically, be predicted since (1) negative emotions can influence cardiac functioning through effects on the autonomic nervous system; (2) several medications prescribed to psychiatric patients influence cardiac function; (3) there is published confirmation of a co-morbidity between depression and cardiovascular disorders; and (4) there is evidence that negative mood states may be a predictor of mortality in patients already diagnosed with coronary heart disease.^9^,^10^ The potential of an augmented risk for cardiovascular incidents in psychiatric patients should be considered when treating such patients.
